# Can Mobile Videocall Assist Laypersons' Use of Automated External Defibrillators? A Randomized Simulation Study and Qualitative Analysis

**DOI:** 10.1155/2020/4069749

**Published:** 2020-10-24

**Authors:** Jun Young Bang, Youngsuk Cho, Gyu Chong Cho, Jongshill Lee, In Young Kim

**Affiliations:** ^1^Department of Emergency Medicine, Kangdong Sacred Heart Hospital, College of Medicine, Hallym University, Seoul, Republic of Korea; ^2^Department of Biomedical Engineering, College of Medicine, Hanyang University, Seoul, Republic of Korea

## Abstract

**Objective:**

To investigate the feasibility of mobile videocall guidance to facilitate AED use by laypersons. *Design, setting, and participants*. A total of 90 laypersons were randomized into three groups: the mobile video call-guided, voice call-guided, and non-guided groups. Participants were exposed to simulated cardiac arrest to use an AED, and guided by video calls, voice calls, or were not guided. We recorded the simulation experiments as a videoclip, and other researchers who were blinded to the simulation assessed the performance according to the prespecified checklist after simulations. *Outcomes measure and analysis*. We compared the performance score and time intervals from AED arrival to defibrillation among the three groups and analyzed the common errors.

**Results:**

There was no significant difference among the three groups in terms of baseline characteristics. Performance scores in the checklist for using AED were higher in the mobile video call-guided group, especially in the category of “Power on AED” and “Correctly attaches pads” than in the other groups. However, the time interval to defibrillation was significantly longer in the mobile video call-guided group.

**Conclusions:**

Mobile video call guidance might be an alternative method to facilitate AED use by laypersons. Therefore, further well-designed research is needed to evaluate the feasibility of this approach in OHCA.

## 1. Introduction

Ventricular fibrillation is one of the major causes of out-of-hospital cardiac arrest (OHCA), and the time from the onset of cardiac arrest to electrical defibrillation is critically related to the survival rate of OHCA patients [1, 2]. The survival rate is reduced by 7–10% for every minute that defibrillation is delayed, and therefore, defibrillation has to be performed as quickly as possible by the layperson at the scene of the cardiac arrest [3–5]. The American Heart Association (AHA) emphasizes the importance of defibrillation programs ((public access defibrillation, PAD) in the cardiopulmonary resuscitation (CPR) guidelines, with recommendations to install automated external defibrillators (AED) in public places for rapid defibrillation and to educate the public to use them [6]. In South Korea, the Act on Emergency Medical Care requires the installation of an automatic defibrillator in a public healthcare institution, ambulance, aircraft, railway vehicles, ships with 20 ton or more capacity, areas with more than 500 households, and in multiuse facilities to enable rapid defibrillation in OHCA patients [7].

Despite these efforts, however, less than 5% of OHCA patients receive defibrillation before the arrival of paramedics following emergency calls to 119 [5]. Lack of information on the location and usage of AEDs and fear of harm to patients are factors that impede the implementation of defibrillation by the public [8]. However, emergency medical dispatcher-guided use of AED is known to resolve this problem [9, 10]. We found that the quality of chest compressions improved when emergency medical dispatchers used video calls to direct CPR as compared to voice calls [11]. However, only a few studies have compared the effects of video calls and voice calls on AED use.

Therefore, we aimed to determine whether instructions via video call helps laypersons to use the AED more accurately than in those via the voice call or in the absence of instructions.

## 2. Methods

### 2.1. Subjects

**Figure 1:**
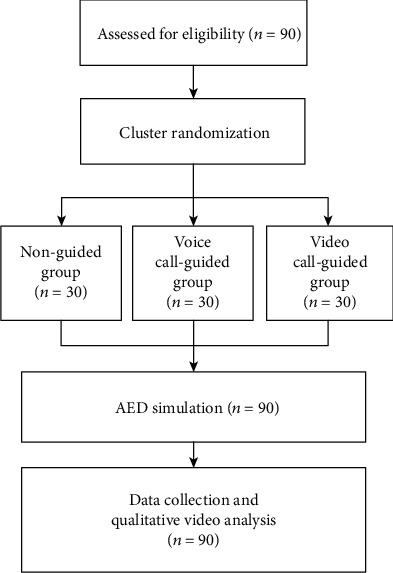
Flow sheet of the study.

This prospective randomized simulation study was conducted to determine whether video call guidance can help a layperson to correctly use the AED in a hypothetical cardiac arrest situation. We enrolled adult college students (age ≥ 18 years) and selected 90 participants who did not have formal training, such as the AHA Basic Life Support (BLS) course, in CPR and AED. In June 2019, we performed a simulation experiment using a mannequin while assuming a cardiac arrest situation. The control group included participants who performed CPR without any guidance (non-guided group), and the two intervention groups were divided into those that received researcher-conducted guidance through a voice call (voice call-guided group) or a video call (mobile video call-guided group). All participants were randomly assigned to three groups and then simulated according to the study protocol (Figure 1). After recording the entire process, the performance was evaluated using a prespecified checklist. Participants provided voluntary written informed consent, after receiving a full explanation of the study's purpose, content, and video recording, before study participation. Those who withdrew from the study or did not participate in the simulation experiment were excluded. In addition, by excluding students who belonged to the college of health and science, we tried to maintain the level of interest for CPR in participants similar to that of the layperson. This study was conducted with the approval of the Institutional Review Board.

### 2.2. Simulation Procedure

The study population was uniformly and randomly assigned to the non-guided, voice call-guided, and video call-guided groups through a computer-based random number program. Before the simulation, participants were blinded as to which group they had been assigned. To reduce the impact of the preceding simulations by other participants, the participants were not permitted to communicate with other participants after the simulation and asked to return home without individual feedback. The simulation operation, data collection, and postassessment process were described in the study protocol based on the preliminary research meeting. The non-guided group had to independently apply the AED to the mannequin under the assumption of an OHCA situation without any questions or guidance during the simulation. The voice call-guided group was instructed to use an AED via a call with a person who was assigned the emergency medical dispatcher's role in a separate room. The participants could communicate with the dispatcher via a voice call, but the dispatcher has standardized to explain only the predefined contents of the script, without sharing any personal opinions. The video call-guided group proceeded similarly as the voice call-guided group, except that the video call group was instructed through a mobile video call. The AED use was recorded with a cellphone camera by another assistant, and the instructor supervised it in real time via video calls. The entire process was filmed, and each process was evaluated with a score by using the performance checklist. The AEDs used in the simulation were educational AEDs (Paramedic CU-ERT®, CU Medical Systems, Korea) where no actual shock was transmitted. After all simulations were completed, all participants received training on the correct use of AEDs.

### 2.3. Data Collection

The simulation process was recorded as a videoclip using a camera. Video recording was performed under controls to ensure that the participants' personal information was not revealed, and the file was saved with a preassigned serial number such that the participants could not identify the assigned group. The performance score evaluation through the checklist was conducted independently by three different emergency medical specialists who did not participate in the simulation; in case of disagreement, the final performance score was confirmed after discussion among the specialists. The main outcome variable was defined as the AED performance of each group. Therefore, the AED checklist of the 2015 AHA Basic Life Support (BLS) course was incorporated as an evaluation tool [12]. AHA's AED checklist comprises five items: “Powers on AED,” “Correctly attaches pads,” “Clears to analysis,” “Clears to safely deliver a shock,” and “Safely delivers a shock and resumes compression.” To evaluate the proficiency of the AED use, the researchers created an evaluation tool (total score, 10 points) by classifying the performance of each item as unsuccessful (0 points), incomplete (1 point), and complete trial (2 points) (supplement 1). Furthermore, the researchers graded the completion rate of the total items to evaluate the global competency. Preliminary experiments were used to analyze the reliability of the evaluation tools, and the intraclass correlation coefficient was estimated to be 0.82 (95% confidence interval (CI), 0.57–0.94). Moreover, through qualitative video analysis, the time interval required for each step such as turning on the power, attaching the pad, pressing the defibrillation button, and restarting chest compression and the total time until the defibrillation were measured as the secondary outcomes. Additionally, the evaluator observed common errors of laypersons using AED in videoclips and self-described them. A survey was conducted on the participants' gender, age, number and contents of CPR training, experience in CPR training within the last 2years, actual experience in using AEDs, and confidence level in using AEDs (rated on a five-point scale).

### 2.4. Statistical Analysis

In the pretest, the average scores of the non-guided, voice call-guided, and video call-guided groups were 6.2, 6.8, and 7.1, respectively. Based on this, 81 samples were deemed necessary to calculate the table fractions with a significance level of 0.05 and a statistical power of 0.8. A total of 90 samples were finalized, factoring in a 10% dropout rate. The analytical method was determined by performing a normality test for each outcome variable. One-way analysis of variance was performed in case of normality among continuous variables, and the Kruskal–Wallis test was used in case of nonnormality test. If necessary, the Mann–Whitney *U* test and Bonferroni calibration were performed with multiple-comparison post hoc tests. Continuous variables are expressed as the mean and standard deviation or median and quartile range, and categorical variables as the frequency and percentage. Statistical analysis was performed using SPSS version 18.0 (SPSS Inc., Chicago, USA), and *p* < 0.05 was considered to indicate statistical significance.

## 3. Results

### 3.1. Participant Characteristics

There were 18 (60%) women in the non-guided group, 19 (63.3%) in the voice call group, and 17 (56.7%) in the video call group, with no significant differences among the three groups (*p* = 0.87). The median age was 24.0, 22.0, and 22.5 years in the non-guided, voice call-guided, and video call-guided groups, respectively, without any significant difference (*p* = 0.07). The experiences of CPR and AED education and the number of education in the last 2 years among the groups were not significantly different (*p* = 0.475, 0.412, and 0.104 in the non-guided, voice call-guided, and video call-guided groups, respectively). Most of the participants had no experience in using real AEDs (Table 1). In addition, the majors of participants were tourism (*n* = 60) and police administration (*n* = 30). But there were no differences in the previous CPR and AED education experiences according to their majors (*p* = 0.290, 0.227, and 0.95, respectively).

### 3.2. Layperson Performance Using AED

The defibrillator performance scores of each group were 4.3 ± 1.21, 5.47 ± 0.94, and 7.47 ± 0.82 in the non-guided, voice call-guided, and video call-guided groups, respectively. The scores in the video call group were significantly higher than those in the other two groups (*p* < 0.001). All participants in the video call group scored 2 points in the “Powers on AED” category compared to the non-guided (0.73 ± 0.91 points) and voice call-guided (1.93 ± 0.37 points) groups. Moreover, the video call-guided group (1.97 ± 0.18) showed a significant difference in the “Correctly attached pad” category than the non-guided (0.77 ± 0.57 points) and voice call-guided (0.73 ± 0.74 points) groups. Both, the non-guided group and voice call-guided groups, only received 1 point in the checklist of “Clears to analysis” and “Clears to safely deliver a shock”; however, the participants in the video call-guided group had scores of 1.3 ± 0.47 and 1.17 ± 0.38 (*p* < 0.001 and 0.005), respectively. Furthermore, the scores in the non-guided and the voice call-guided groups were 0.8 ± 0.41 in the “Safely delivered a shock and resumed compression,” although the score in the video call-guided group was 1.03 ± 0.12 (*p* = 0.017) (Table 2). To evaluate whether the AED checklists were completed, we compared their completion rates. The video call-guided group was significantly higher than the non-guided group and the voice call group (*p* < 0.001).

### 3.3. Time Interval and Qualitative Video Analysis

**Figure 2:**
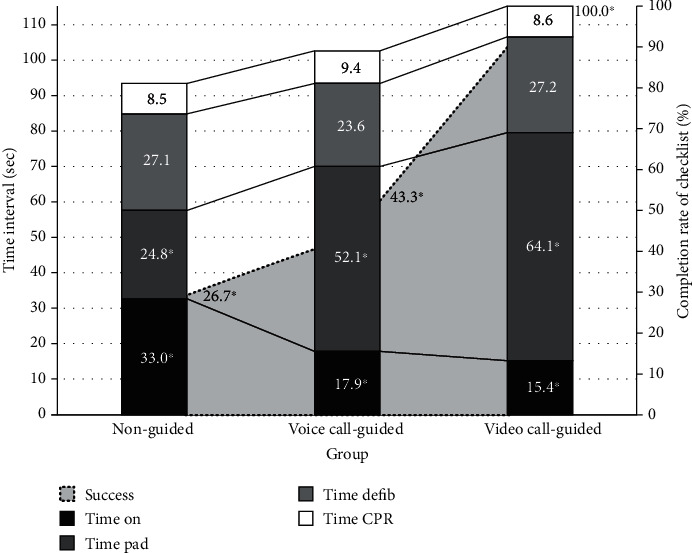
Analysis of time interval for using AED. Black bar, time interval to power on AED; dark gray bar, time interval to attach pads on the manikin; light gray bar, time interval to deliver shock; white bar, time interval to resume chest compression; AED, automated external defibrillator; gray-colored area under the graph, total completion rate for AED checklist. ^∗^ means statistically significant among the three groups, *p* < 0.05.

The total time to defibrillation was significantly shorter in the non-guided group than in the voice call-guided and video call-guided groups (84.2 ± 28.85 s vs. 93.57 ± 20.63 s vs. 106.7 ± 26.9 s; *p* = 0.004). The time taken to power on was significantly longer in the non-guided group (32.97 ± 26.96 s vs. 17.9 ± 8.48 s vs. 15.4 ± 5.49 s, *p* < 0.001) than in the other groups, whereas the time from powering on to attaching the pads was significantly shorter in the non-guided group (24.83 ± 31.44 s vs. 52.1 ± 16.0 s vs. 64.07 ± 22.76 s, *p* < 0.001) (Figure 2). The common errors in AED use included not clearing other people before delivering a shock (85/90, 94.4%), not removing the pad cover (25/99, 27.8%), and not applying the pad on the correct place (22/90, 24.4%). Moreover, there were instances where the pad cable was not connected (11/90, 12.2%), chest compression was not performed promptly after defibrillation (11/90, 12.2%), and the AED was not powered on first (5/90, 5.6%). In general, confusion in the left and right directions with regard to the mannequin (9/90, 10.0%) and inability to hear the dispatcher's advice due to noise (1/90, 1.1%) were noted as miscellaneous factors (Table 3).

## 4. Discussion

The participants who did not have formal training were included in this study. However, in the results, more than 70% of the participants had an experience of CPR education and more than 20% has AED education. In Korea, CPR education has been mainly conducted in school, military, workplace, etc. Furthermore, men were educated CPR at least once during compulsory military service. But this type of education was frequently done as a lecture form without practice sessions, in a large group, in a short time, and using only video clips or slides. Because the participants remember such an experience, they might answer like that way.

The overall performance score of the AED simulation ranged from high to low in the video call-guided, voice call-guided, and non-guided groups. In the post hoc analysis, both the voice and video call-guided groups showed higher scores in the category of “Power on AED,” and the video call-guided group showed significantly higher scores than the other two groups in the category “Correctly attaches pad.” Despite significant intergroup differences in the categories of “Clears to analysis,” “Clears to safely deliver a shock,” and “Safely delivers a shock and resumes compression,” there was no significant difference in pairwise comparisons between the two groups. In a meta-analysis, the proportion of laypersons who knew how to correctly use an AED was reported to be 7-26% [12]. In this study, the non-guided group received a lower performance score than the other groups, which was consistent with the results of the meta-analysis.

The score of the non-guided group in the category “Powers on AED” was relatively low, but the difference between the voice call and video call-guided groups was not significant, which may be because pressing the power button is an easy step to follow in the dispatcher's instruction. In contrast, this may be a difficult step for laypersons unfamiliar with AED use. The step of attaching the pads to the correct position could be performed more precisely in the video call-guided group because the dispatcher could direct the laypersons through real-time interactive communication. To date, few clinical studies have evaluated whether AED pad placement in the right location actually affects the survival rate and recovery of spontaneous circulation in OHCA patients. However, some animal studies have reported that maximizing the current through the myocardium by correctly attaching the pads is critical for successful defibrillation [13–16]; therefore, video calls are helpful for correct pad attachment and are thus associated with successful defibrillation. Moreover, as the “Clears to analysis,” “Clears to safely deliver a shock,” and “Safely delivers a shock and resumes compression” steps were guided by a prerecorded voice comment for the AED, these might be less affected by voice and video calls.

The total time to AED use was the longest in the video call-guided group and the shortest in the non-guided group. Thus, as the cessation of chest compression is inevitable when using a defibrillator, it is important to perform defibrillation as soon as possible [17, 18]. Although the video call-guided group had the longest time to defibrillate, none of the three groups exceeded the recommended time for AED use [19]. Rather, in terms of completeness of all checklists, the non-guided group had a score of 26.7%, which showed a poorer performance than the other groups (43.3% and 100%). The time taken to power on was shortest in the video call-guided group. However, the time difference was not due to the speed of the voice call-guided group, but rather, due to the failure to properly understand the powering on of the AED in the nonguided group. The first step of AED use, powering on, is very important because the voice guidance guides the pad attachment, electrocardiogram analysis, and defibrillation instructions. Video calls have a huge advantage of being helpful in the first step. In fact, some defibrillators that are currently available can automatically power on when the cover is opened, or the power button can be easily and clearly indicated to reflect the purpose. In contrast, the time taken to attach the pad in the video call-guided group was significantly longer than that in the control group, which might be attributed to the time expended in following the emergency medical dispatcher's instruction and correction of the pad placement errors in real time. A large number of participants in the voice call and video call-guided group were instructed or corrected pad placement by dispatchers, communicating interactively (21/30 in the voice call group, 30/30 in the video call group).

In the qualitative video analysis, most of the participants in the groups did not ensure that no one was touching the person before delivering a shock. However, some participants did not know how to remove the AED pad cover or how to power on the AED, possibly because they had no experience in the stepwise education for AED practice. To reduce the abovementioned errors, CPR training, including not only the lecture but also AED practical training, is needed. Furthermore, laypersons could not competently use the AED in accordance with the dispatcher's instruction despite being in the voice call-guided group, possibly because they did not understand the anatomical directional terms or hear the advice clearly because of ambient noise. Accordingly, the dispatcher should also consider the possibility of communication errors between dispatchers and laypersons, and video call guidance could be an alternative method to facilitate AED use in CPR.

This study has several limitations. First, because it is a relatively small study of university students in a specific area, it cannot represent the general characteristics, such as age, regional distribution, and accessibility of video calls, of the general public, and the results may not be easily generalizable. Second, this is a simulation study that dealt only with AED use for the virtual OHCA situation, which can be quite different from the actual OHCA situation. During actual CPR, the chest compression quality and minimizing of interruptions are important [20, 21]. However, this study did not fully consider factors other than the time required for defibrillation. Third, there was no consideration of communication errors, poor quality of video calls, and the actual problem of showing video when CPR was performed by the caller. Fourth, it is known that a variety of complex factors affect the low defibrillation enforcement rate in the general public; therefore, it may be difficult to improve the actual use of AEDs based only on such educational guidance. Therefore, a large-scale prospective clinical study is needed to determine whether guidance on using AEDs through video calls is effective in OHCA.

## 5. Conclusion

In summary, this simulation evaluation confirmed that the AED performance of the laypersons improved in the video call-guided group than in the control or voice call-guided group. When using an AED alone, the AED could not be turned on quickly and the pad could not be properly placed; therefore, a video call could be considered as a feasible alternative in layperson CPR for OHCA.

## Figures and Tables

**Table 1:** Basic characteristics of participants (*n* = 90).

Variables	Group			*p*
	Non-guided (*n* = 30)	Voice call-guided (*n* = 30)	Video call-guided (*n* = 30)	
Gender, female	18 (60.0)	19 (63.3)	17 (56.7)	0.870
Age, year	24.0 (23.0-25.0)	22.0 (22.0-23.0)	22.5 (22.0-26.25)	0.070
Experience of				
CPR education	23 (76.7)	21 (70.0)	25 (83.3)	0.475
CPR education including AED	8 (26.7)	7 (23.3)	12 (40.0)	0.412
Number of CPR education in recent 2 years	0.0 (0.0-1.0)	0.0 (0.0-1.0)	1.0 (0.0-1.3)	0.104
Real AED use	2 (6.7)	0 (0.0)	3 (10.0)	0.227

Categorical variables were expressed by *n*(%), continuous variables by median (interquartile range). Statistical analysis was conducted by nonparametric test. CPR: cardiopulmonary resuscitation; AED: automated external defibrillator.

**Table 2:** Comparison of performance in AED simulation test among groups.

	Group			*p*
	Non-guided (*n* = 30)	Voice call-guided (*n* = 30)	Video call-guided (*n* = 30)	
Total score (10 points)	4.3 ± 1.21	5.47 ± 0.94	7.47 ± 0.82	<0.001
Powers on AED (2 points)	0.73 ± 0.91	1.93 ± 0.37	2.0 ± 0.0	<0.001
Correctly attaches pads (2 points)	0.77 ± 0.57	0.73 ± 0.74	1.97 ± 0.18	<0.001
Clear for analysis (2 points)	1.0 ± 0.0	1.0 ± 0.0	1.3 ± 0.47	<0.001
Clear to safely deliver a shock (2 points)	1.0 ± 0.0	1.0 ± 0.0	1.17 ± 0.38	0.005
Safely delivers a shock and resume compression (2 points)	0.8 ± 0.41	0.8 ± 0.41	1.03 ± 0.12	0.017
Completeness for AED checklist	8 (26.7)	13 (43.3)	30 (100.0)	<0.001
Time interval from AED arrival to defibrillation (sec)	84.2 ± 28.85	93.57 ± 20.6	106.7 ± 26.9	0.004

Categorical variables were expressed by *n*(%), continuous variables by mean (standard deviation). Statistical analysis was conducted by nonparametric test. AED: automated external defibrillator.

**Table 3:** Common errors observed in the video clips.

Common errors	Non-guided (*n* = 30)	Voice call-guided (*n* = 30)	Video call-guided (*n* = 30)	Total (*n* = 90)
“Layperson did not power on AED first.”	5	0	0	5 (5.6%)
“Layperson did not remove the pads cover.”	8	17	0	25 (27.8%)
“Layperson did not apply the pad on the right place.”	13	8	1	22 (24.4%)
“Layperson did not connect the pad cable.”	9	2	0	11 (12.2%)
“Layperson did not clear other people before delivering a shock.”	30	30	25	85 (94.4%)
“Layperson did not perform chest compression promptly after defibrillation.”	4	7	0	11 (12.2%)
“Layperson confused about the left and right direction to dispatcher's advice.”	0	5	4	9 (10.0%)
“Layperson did not hear the advice due to noise.”	0	1	0	1 (1.1%)

AED: automated external defibrillator.

## Data Availability

The data used to support the findings of this study have not been made available because participants' privacy should be protected.

## Supplementary Materials

Supplementary Materials Supplement 1. AED Skill Testing Checklist.
