# Supplementary figures and images for: Can Mobile Videocall Assist Laypersons' Use of Automated External Defibrillators? A Randomized Simulation Study and Qualitative Analysis
Source: Biomed Res Int. 2020 Oct 24;2020:4069749. doi: 10.1155/2020/4069749 (PMC7604583; doi:10.1155/2020/4069749)

**Supplement 1. AED Skill Testing Checklist**


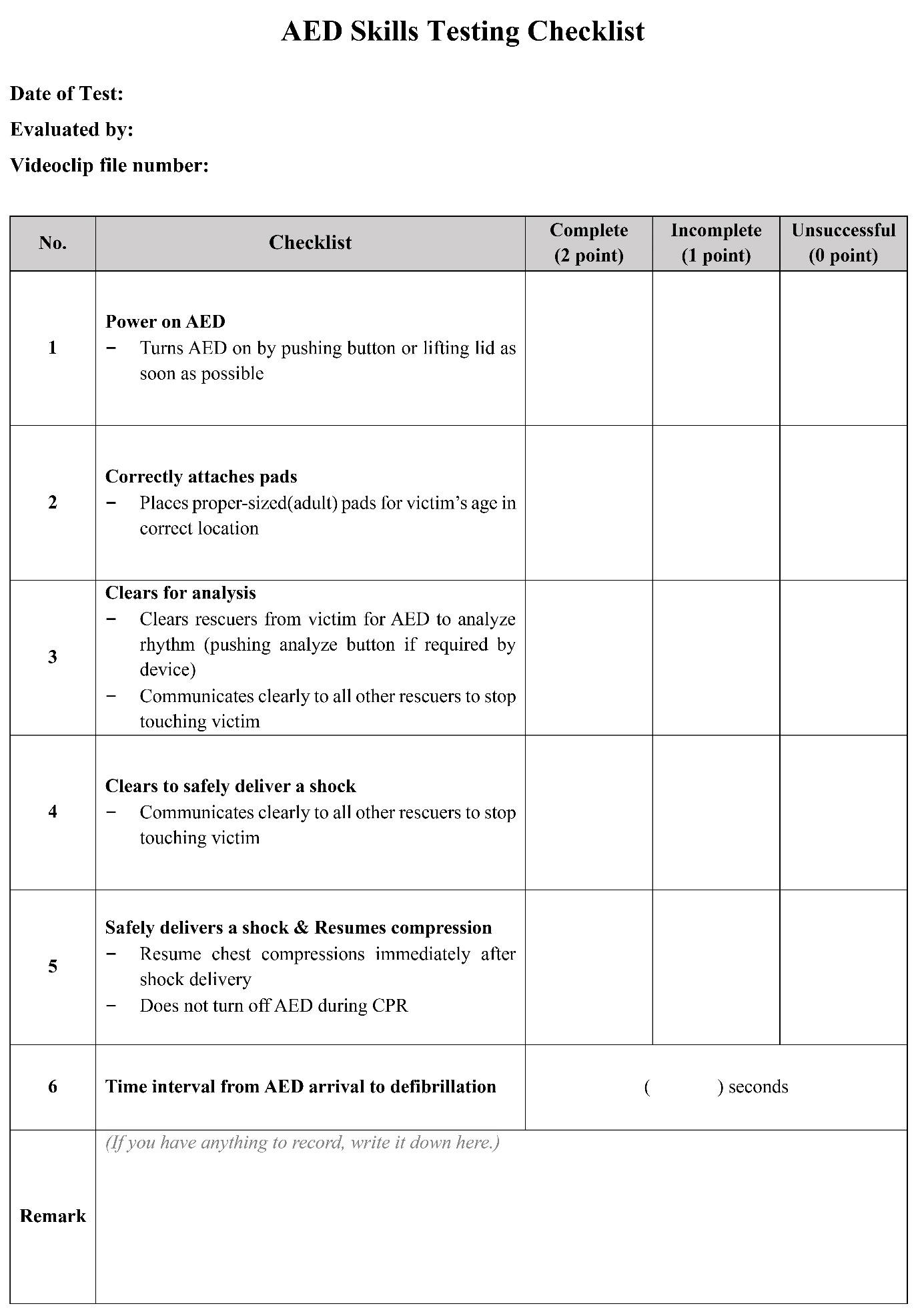

Supplement: Supplementary Materials — Supplement 1. AED Skill Testing Checklist. [file 4069749.f1.docx]
